# Analysis of particle therapy registries based on a unified treatment policy for esophageal cancer

**DOI:** 10.1093/jrr/rrad012

**Published:** 2023-04-07

**Authors:** Sunao Tokumaru, Hitoshi Ishikawa, Toshinori Soejima, Takuya Kimoto, Yosuke Takakusagi, Hiroyasu Tamamura, Hitoshi Wada, Hiroshi Taguchi, Yusuke Uchinami, Yuichi Hiroshima, Hidehiro Hojo, Takashi Kamei, Manabu Muto, Masataka Igeta

**Affiliations:** Department of Radiology, Hyogo Ion Beam Medical Center, 1-2-1 Kouto, Shingu-cho, Tatsuno, Hyogo 679-5165, Japan; National Institutes for Quantum and Radiological Science and Technology, QST Hospital, 4-9-1 Anagawa, Inage-ku, Chiba 263-8555, Japan; Department of Radiation Oncology, Hyogo Ion Beam Medical Center Kobe Proton Center, 1-6-8 Minatojima Minamimachi, Chuo-ku, Kobe, Hyogo 650-0047, Japan; Department of Radiology, Kyoto Prefectural University of Medicine, Kawaramachi-Hirokoji, Kamigyo-ku, Kyoto 602-8566, Japan; Department of Radiation Oncology, Kanagawa Cancer Center, 2-3-2 Nakao, Asahi-ku, Yokohama, Kanagawa 241-8515, Japan; Proton Therapy Center, Fukui Prefectural Hospital, 2-8-1, Yotsui, Fukui, Fukui 910-8526, Japan; Department of Radiation Oncology, Southern TOHOKU Proton Therapy Center, 7-172, Yatsuyamada, Fukushima, Koriyama 963-8052, Japan; Department of Radiation Oncology, Hokkaido University Hospital, Kita 15, Nishi 7, Kita-ku, Sapporo, Hokkaido 060-0808, Japan; Department of Radiation Oncology, Faculty of Medicine, Hokkaido University, Kita14, Nishi5, Kita-Ku, Sapporo, Hokkaido 060-8648, Japan; Radiation Oncology & Proton Medical Research Center, Faculty of Medicine, University of Tsukuba, 2-1-1, Amakubo, Tsukuba, Ibaraki 305-8576, Japan; Department of Radiation Oncology, National Cancer Hospital East, 6-5-1, Kashiwanoha, Kashiwa, Chiba 277-8577, Japan; Department of Surgery, Graduate School of Medicine, Tohoku University, 1-1, Seiryomachi, Aoba-ku, Sendai, Miyagi 980-8574, Japan; Department of Therapeutic Oncology, Graduate School of Medicine, Kyoto University, 53 Shougoin Kawaharachou, Sakyo-ku, Kyoto 606-8507, Japan; Department of Biostatistics, Hyogo College of Medicine, 1-1, Mukogawacho, Nishinomiya, Hyogo 663-8501, Japan

**Keywords:** esophageal cancer, particle therapy, outcome, toxicity

## Abstract

This study aimed to evaluate the efficacy and safety of particle therapy (proton beam therapy and carbon-ion radiotherapy) for esophageal cancer by analyzing prospective nationwide registry data from particle therapy facilities throughout Japan. Patients diagnosed with esophageal cancer who received particle therapy between May 2016 and June 2018 were recruited from the registries of 12 particle therapy centers in Japan. Eventually, we enrolled 174 patients who met the inclusion criteria. Of the 174 patients, 137 (78.7%) were male, with a median age of 69 years (range: 41–88 years). Clinical stages included I (*n* = 55; 31.6%), II (*n* = 31; 17.8%), III (*n* = 82; 47.1%), IV (*n* = 3; 1.7%) and unknown (*n* = 3; 1.7%) (Union for International Cancer Control, seventh edition), and the median follow-up period was 908 days (range: 76–1669 days) for all patients. The 3-year overall survival (OS) rate, the 3-year progression-free survival (PFS) rate and the 3-year local control (LC) rates were 60.5, 53.2 and 72.7%, respectively. For each clinical stage, the 3-year OS rates were I, 84.8%; II, 60.3% and III, 42.9%; the 3-year PFS rates were I, 71.9%; II, 58.3% and III, 37.0% and the 3-year LC were I, 78.4%; II, 79.8% and III, 65.2%, respectively. Notably, four patients (2.3%) with ≥Grade 3 cardiopulmonary toxicities were observed (Common Terminology Criteria for Adverse Events, version 5.0). Our study showed that particle therapy for esophageal cancer has lower rates of adverse cardiopulmonary events than X-ray radiotherapy.

## INTRODUCTION

According to the International Agency for Research on Cancer, esophageal cancer is the sixth leading cause of cancer-related deaths worldwide [1]. The Registration Committee for Esophageal Cancer of the Japan Esophageal Society also reported that esophageal cancer is the eighth most common cause of cancer-related deaths in Japan [2]. In Japan, squamous cell carcinoma accounted for 87.8% of all esophageal cancer cases in 2013 [2], and with an aging society, an increasing number of elderly patients are being diagnosed with esophageal cancer, of which 44.1% of them are aged 70 years and above [2].

Presently, surgery remains the first choice of treatment for operable esophageal cancer in Japan, with 61.2% of the affected patients undergoing treatment [2]. Nevertheless, clinical trials looking at the efficacy and toxicity of chemoradiotherapy (CRT) in treating esophageal cancer have also been reported [3–8]. In particular, 15.1% of patients with esophageal cancer in Japan reportedly received CRT as a primary treatment in 2013 [2]. Besides, in clinical guidelines, CRT is considered the standard treatment for inoperable locally advanced esophageal cancer [9, 10].

Proton beam therapy (PBT) and carbon-ion radiation therapy (CIRT) are types of the particle therapy. Particle therapy delivers concentrated doses of protons or carbon-ion beams to target tissues with minimal harm to surrounding normal tissues, compared with conventional X-ray radiotherapy or intensity-modulated radiotherapy (IMRT) [11–13].

Therefore, this study aimed to evaluate the efficacy and safety of particle therapy. It is worth noting that an increasing number of facilities have recently become available for particle therapy. Moreover, this Japanese Nationwide Registry Study on particle therapy for esophageal cancer was initiated in 2016, and all institutions that performed particle therapy for patients with esophageal cancer than in Japan were also included in this study.

## MATERIALS AND METHODS

### Study design and patients

This study was a multicenter, prospective observational registry conducted by Proton-Net (a Japanese PBT network) and the Japan Carbon-ion Radiation Oncology Study Group (J-CROS). Proton-Net is a study group that includes all 19 PBT institutions, and J-CROS is a study group that includes all 7 CIRT institutions currently in Japan. About 12 facilities (PBT = 10, CIRT = 2) participated in this multi-institutional study on esophageal cancer. This study was approved by the Esophageal Cancer Working Group of the Particle Beam Therapy Committee and Subcommittee of the Japanese Society for Radiation Oncology (JASTRO). Written informed consent was obtained from the patients.

Between May 2016 and June 2018, patients diagnosed with esophageal cancer and treated with particle therapy, with or without chemotherapy, absence of metastases to distant organs and no other sites with cancer were recruited from the prospective databases of particle therapy facilities in Japan. The clinical stage was evaluated using the TNM Classification of Malignant Tumors, seventh edition [14].

### Treatment

Particle beam therapy for esophageal cancer is performed (as an advanced medical treatment) in accordance with the unified treatment policy established by JASTRO [15]. Dose fractionation was performed according to the unified treatment policy, and specifically, in PBT, these values range from 60 Gy (relative biological effectiveness [RBE])/30 fractions to 70 Gy (RBE)/35 fractions for stages I–VI esophageal cancer, in CIRT, these values range from 48 Gy (RBE)/12 fractions to 50.4 Gy (RBE)/12 fractions for stage I esophageal cancer [15]. When these are converted to the biological effective dose (BED) with an alpha/beta ratio of 10, using a linear-quadratic model as follows: Gy (RBE) BED_10_ = total dose × (1 + dose per fraction/10), these values range from 67.2 to 84.0 (Gy (RBE) BED_10_). Gross tumor volume (GTV) was defined as the primary tumor and lymph node metastasis based on endoscopy and imaging. Typically, the clinical target volume (CTV) was defined as GTV obtained by adding 2–5 cm margins to both cranial and caudal edges and 0.5–2 cm margins from other directions, the planning target volume was defined as the CTV plus 0.5–1 cm. The elective nodal irradiation (ENI) field usually depends on the location of the primary esophageal cancer and the institution’s treatment decisions. For example, ENI can be performed either from the bilateral supraclavicular lymph node to the celiac artery or from the aortic arch to the peri-gastric region, etc. Moreover, due to the narrow field of particle therapy, the use of X-ray radiotherapy in ENI is acceptable. Importantly, in the unified treatment policy of PBT [15], concurrent chemotherapy can be used for esophageal cancer in accordance with clinical practice guidelines.

### Statistical analysis

Patient characteristics and radiation therapy were initially summarized. The median, minimum and maximum values were calculated for the continuous variables, and frequency distributions were shown for the categorical data. Overall survival (OS) was defined as the time from the start of particle therapy to the last follow-up date or death. Progression-free survival (PFS) was defined as the time from the start date of particle therapy to the last follow-up date, recurrence or exacerbation of esophageal cancer or death. For OS and PFS, the cumulative survival rates at 3 years were calculated using the Kaplan–Meier method. Local control (LC) was measured from the date of initial particle therapy to the date of the first local recurrence in the irradiated area or the date of the last follow-up. For LC, we estimated the cumulative incidence functions at 3 years and documented mortality before local recurrence as a competing risk. Then, in addition to the limitations of direct comparisons between different trials, OS and PFS were compared with OS and PFS reported from photon therapy [3, 16, 17] using a *Z*-test based on double-logarithmic transformation for exploratory purposes. All statistical analyses were performed using SAS (ver. 9.4, SAS Institute, Cary, NC, USA) and R 4.2.1 [18].

### Evaluation of safety

Adverse events of Grade 3 or higher were assessed according to the Common Terminology Criteria for Adverse Events (CTCAE) v5.0 [19].

### Selection of literature to be compared (systematic review)

**Fig. 1:**
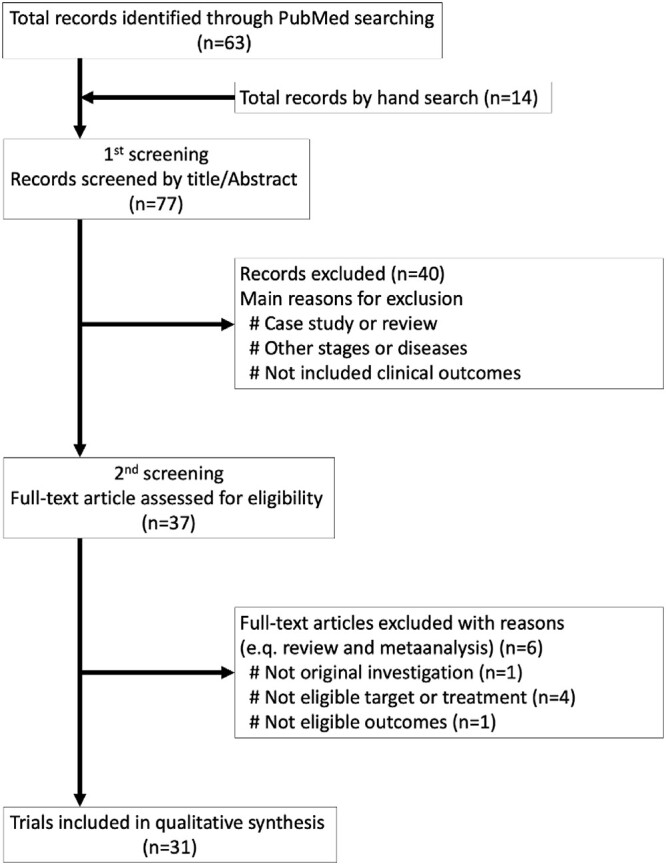
Shows the flow diagram of literature selection.

The systematic review was performed in accordance with the Preferred Reporting Items for Systematic Review and Meta-Analyses guidelines [20]. English documents from 2000 to 2021 were extracted using the following search formula (mainly comparing particle therapy and X-ray radiotherapy) from PubMed, and added important papers on (chemo)radiation therapy as a hand search, referring to the description of the clinical practice guidelines for esophageal cancer [21, 22] before being narrowed to 31 studies after two rounds of screening. The process of adoption is shown in Figure 1. After excluding reports with a small number of cases and reports that overlapped with other reports, 11 studies describing clinical results were selected [3–8,16,17,23–25] (Table 1).

**Table 1:** Summary of previous reports

Author	Study design	Modality	*N*	Stage	OS (%)	PFS (%)	Cardiac toxicity ≥ G3 (%)	Pulmonary toxicity ≥ G3 (%)
Year								
Lin [16]	Randomized- Phase II	IMRT	61	I-III	50.8 (3Y)	44.5 (3Y)	23	
2020		PBT	46		51.2 (3Y)	44.5 (3Y)	13	
Fang [17]	Propensity score matching	IMRT	66	III-IV	38 (3Y)	22 (3Y)	NA	
2018		PBT	67		47 (3Y)	41 (3Y)		
Xi [25]	Retrospective	IMRT	211	I-III	31.6 (5Y)	20.9 (5Y)	7.1	
2017		PBT	137		41.6 (5Y)	34.9 (5Y)	3.0	
Wang X [27]	Retrospective	IMRT	320	I-III	NA	NA	18 (2Y)/ 21 (5Y)	
2020		PBT	159				11 (2Y)/ 13 (5Y)	
Tahara [3]	Phase I/II	3DCRT	44	II-IV	all: 65 (3Y)	NA	2.3	2.3
2015					PII: 61.9 (3Y)			
Shinoda [4]	Randomized- Phase II	2D-3DCRT	71	II-IV	25.0 (3Y)	NA	3	9
2015			72		25.5 (3Y)		0	9
Kato [5]	Phase II	2D-3DCRT	76	II-III	47.1 (3Y)	32.9 (3Y)	16	9
2011								
Nishimura [6]	Phase III	2D-3DCRT	46	II-IV	39^a^ (3Y)	NA	11.9	9.5
2009			45		32^a^ (3Y)		6.7	6.7
Kato [7]	Phase II	2D-3DCRT	72	I	80.5 (4Y)	60.8 (4Y)	1.4	2.8
2009								
Minashi [8]	Phase III	3DCRT + endo-scopic treatment	96	I	90.7^a^ (3Y)	NA	2.1	1.0
2019								
Ono [23]	Retrospective	PBT	202	I-IV	66.7 (3Y)	32.9 (3Y)	1	0.5
2019								
This study	Prospective	PBT, CIRT	174	I-IV	60.5 (3Y)	53.2 (3Y)	1.7	0.6

^a^Estimated value, Abbreviations; OS: overall survival, PFS: progression-free survival, IMRT: intensity-modulated radiotherapy, PBT: proton beam therapy, 3D-CRT: three-dimensional conformal radiotherapy, 2D: two-dimensional (radiotherapy), CIRT: carbon-ion radiation therapy, PII: phase II.

The search formula:

#1 AND #2 AND #3.

#1 “esophageal cancer”[TIAB].

#2 “cancer”[TIAB] “cancers”[TIAB] OR “neoplasm”[TIAB] “neoplasms”[TIAB] OR “neoplasms”[MH] OR “tumor”[TIAB] OR “tumors”[TIAB] OR “tumor”[TIAB] OR “carcinoma”[TIAB] OR “carcinomas”[TIAB] (English, from 2000 to 2021).

#3 “proton therapy”[Title/Abstract] OR “proton radiotherapy” [Title/Abstract] OR “proton beam therapy”[Title/Abstract] OR “proton beam radiotherapy”[Title/Abstract] OR “carbon ion therapy” [Title/Abstract] OR “carbon ion radiotherapy”[Title/Abstract] OR “carbon ion beam therapy”[Title/Abstract] OR “carbon ion beam radiotherapy”[Title/Abstract] OR “heavy ion radiotherapy”[Title/Abstract] OR “heavy ion radiotherapy”[MeSH Terms] OR “hadron therapy”[Title/Abstract] OR “hadrontherapy”[Title/Abstract].

## RESULTS

### Patient characteristics

One hundred and seventy-four patients fulfilled the inclusion criteria. The baseline characteristics of the cohort are summarized in Table 2. Of the 174 patients, 137 (78.7%) were male, with a median age of 69 years (range: 41–88 years). Clinical stages included I (*n* = 55; 31.6%), II (*n* = 31; 17.8%), III (*n* = 82; 47.1%), IV (*n* = 3; 1.7%) and unknown (*n* = 3; 1.7%). Notably, 91 (52.3%) patients had lymph node metastases, 160 (92.0%) had squamous cell carcinoma and only 5 (2.9%) had adenocarcinoma. In addition, 169 (97.1%) patients were with PS (performance status) 0 or PS 1, and no patients were with PS 3 or higher.

**Table 2:** Patient and treatment characteristics

Characteristics	*N*	%
Age (y): median 69 (range: 48–88)		
Sex		
F	37	21.3
M	137	78.7
Performance Status		
PS0	112	64.4
PS1	57	32.8
PS2	5	2.9
PS3-4	0	0
Clinical stage		
I	55	31.6
II	31	17.8
III	82	47.1
IV	3	1.7
Unknown	3	1.7
T stage		
T1	56	32.2
T2	19	10.9
T3	63	36.2
T4	33	19.0
Unknown	3	1.7
N stage		
N0	76	43.7
N1-3	98	56.3
Pathology		
Squamous cell carcinoma	160	92.0
Adenocarcinoma	5	2.9
Other	5	2.9
Unknown	4	2.3
Tumor location		
Cervical	22	12.6
Thoracic	151	86.8
Abdominal	1	0.6
Radiation therapy		
Total dose: median (Gy (RBE) BED10): 79.2 (range: 57.6-91.2)		
Particle therapy		
Proton beam therapy	163	93.7
Carbon-ion radiation therapy	11	6.3
Elective nodal irradiation		
Yes: using particle therapy	42	24.1
Yes: using X-ray radiotherapy	53	30.5
No	79	45.4
Concurrent chemotherapy		
Yes	118	67.8
No	56	32.2

Abbreviations; RBE: relative biological effectiveness, BED: biological effective dose

Scheduled particle therapy was performed in all except two patients, and their median total BED_10_ was 79.2 Gy (RBE) (range: 57.6–91.2 Gy [RBE]). ENI was performed in 95 (54.6%) patients, and 118 (67.8%) patients underwent concurrent chemotherapy at 8 PBT centers. In particular, the most commonly used chemotherapy regimens were cisplatin and 5-fluorouracil (101/118).

### Survival

The median follow-up time was 908 days (range: 76–1669 days). From the data collected, we found that 67 patients died; among them, 52 died from esophageal cancer and 15 from other causes. In addition, recurrence was observed in 71 patients, and the sites of recurrence were local (irradiated site) in 36 cases, lymph nodes in 14 cases and distant metastasis in 25 cases, including duplication.

**Fig. 2:**
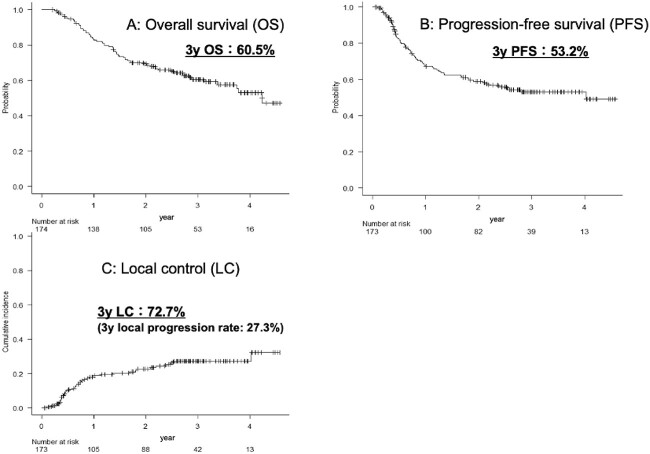
Shows the OS curve (**A**) and the PFS curve (**B**), and cumulative local recurrence incidence (**C**) in all cases.

**Fig. 3:**
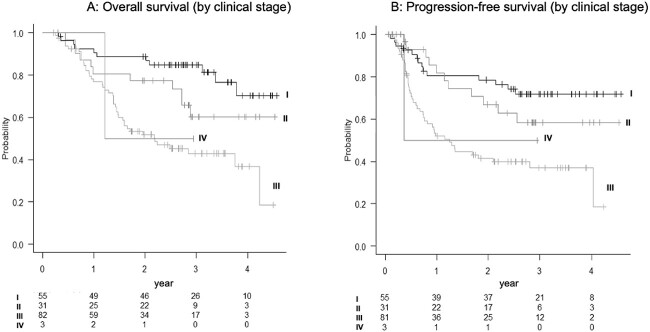
Shows the OS curve (**A**) and the PFS curve (**B**) by clinical stage.

The 3-year OS rate was 60.5% (95% confidence interval [CI]: 52.2–67.9%) (Fig. 2A), the 3-year PFS rate was 53.2% (95% CI: 44.7–61.0%) (Fig. 2B) and the 3-year LC rate was 72.7% (95% CI: 65.0–80.0%) (Fig. 1C), respectively. The 3-year OS rates by clinical stage were 84.8% for stage I, 60.3% for stage II and 42.9% for stage III (Fig. 3A); the 3-year PFS rates by clinical stage were 71.9% for stage I, 58.3% for stage II and 37.0% for stage III (Fig. 3B) and the 3-year LC rates by clinical stage were 78.4% for stage I, 79.8% for stage II and 65.2% for Stage III.

We selected three articles [3,16,17] in which patients were treated with X-ray radiotherapy using IMRT or 3D conformal radiotherapy and recorded their OS. Patients with the same clinical stage (e.g. stages I–III) as those in the three publications were selected from our particle therapy cohort, and their OS was calculated. The 3-year OS rate of each article and the 3-year OS rate of each patient selected in our particle therapy registry were compared using the *Z*-test based on a double-logarithmic transformation (Table 3). Likewise, two papers reporting PFS were selected [16, 17], and the 3-year PFS rate of each paper and the 3-year PFS rate of each patient selected in our particle therapy registry were compared using the *Z*-test (Table 3). Notably, a negative *Z*-value is better for particle therapy.

**Table 3:** Comparison of X-ray radiotherapy and this study (particle therapy)

	X-ray radiotherapy			Particle therapy			*Z* test	
Author	*N*	Stage: *N*	OS (3Y)	*N*	Stage: *N*	OS (3Y)	*Z*	*P*
Lin [16]	61	I:4, II:24, III:33	50.8%	171	I:55, II:31, III:82	60.1%	−2.147	0.0318
Fang [17]	66	III:62, IV:4	38%	85	III:82, IV:3	43.2%	−0.870	0.3842
Tahara [3]	44	II:26, III:17, IV:1	65%	116	II:31, III:82, IV:3	47.8%	3.691	0.0002
Author	N	Stage: *N*	PFS (3Y)	*N*	Stage: *N*	PFS (3Y)	*Z*	*P*
Lin [16]	61	I:4, II:24, III:33	44.5%	171	I:55, II:31, III:82	53.0%	−1.923	0.0545
Fang [17]	66	III:62, IV:4	22%	85	III:82, IV:3	37.5%	−2.642	0.0083

*Z*: Negative value indicates better particle therapy, Abbreviations; OS: overall survival, PFS: progression-free survival, *P*: *P*-value

### Adverse events

Grade 3 or higher adverse events assessment using CTCAE v5.0 is shown in Table 4. Cardiopulmonary adverse events of Grade 3 or higher were pericardial effusion in two cases, pericarditis in one case and one radiation pneumonitis case, with a total incidence of 2.3%. Among the cases with Grade 3 pericardial effusions, one patient had a case in which the heart was not irradiated; as a result, this case was not considered to be related to particle therapy. Adverse events in the esophagus of Grade 3 or higher included esophagitis in five cases, esophageal ulcer in two cases, esophageal perforation in two cases, esophageal stricture in two cases and esophageal fistula in one case. Of these, one (0.6%) patient died of complications and was classified as Grade 5.

**Table 4:** Adverse events (Grade 3 or higher)

Location	Events	Grade 3	Grade 4	Grade 5	Total
		*N* (%)	*N* (%)	*N* (%)	*N* (%)
Esophagus	Esophagitis, esophageal ulcer, esophageal fistula, esophageal stenosis	9 (5.2)	2 (1.1)	1^a^ (0.6)	12 (6.9)
Heart	Pericardial effusion, pericarditis	2^b^ (1.1)	0	1^a^ (0.6)	3 (1.7)
Lung	Pneumonitis	1 (0.6)	0	0	1 (0.6)
Skin	Dermatitis	2 (1.1)	0	0	2 (1.1)
Pharyngolarynx	Hoarseness, pharyngolaryngeal pain	2 (1.1)	0	0	2 (1.1)
Spine	Purulent spondylitis	1 (0.6)	0	0	1 (0.6)
Whole body	Anorexia	1 (0.6)	0	0	1 (0.6)
Bone marrow	Neutrophil count decreased, white blood cell count decreased	2 (1.1)	0	0	2 (1.1)
Stomach	Gastric perforation	0	1^c^(0.6)	0	1 (0.6)

^a^Same case.

^b^One case had no causal relationship with particle therapy.

^c^No causal relationship with particle therapy.

## DISCUSSION

We have evaluated the safety and efficacy of particle therapy for the treatment of esophageal cancer using prospective data from Japan. To our knowledge, this is the first study to investigate a prospective multicenter cohort of patients undergoing particle therapy (both PBT and CIRT) for esophageal cancer worldwide.

In a previous retrospective analysis of PBT at four centers in Japan from 2009 to 2013 [23], the 3-year OS rate for all cases was 66.7%, and the 3-year OS rates for each clinical stage were 84% for stage I, 71% for stage II and 59% for stage III, whereas the 3-year PFS rate for all cases was 32.9%. Compared with the present study, the 3-year OS rate for all cases recorded was 60.5%, and the 3-year OS rate by clinical stage was 84.8% for stage I, 60.3% for stage II and 42.9% for stage III, whereas the 3-year PFS rate for all cases was 53.2%. Therefore, we considered that the survival rate reproduced the results of PBT reported in Japan. However, there are no reports of clinical outcomes in patients with esophageal cancer treated with CIRT from multiple institutes.

Particle therapy delivers concentrated doses of proton beams or carbon-ion beams to target tissues with minimal harm to surrounding normal tissues than conventional X-ray radiotherapy or IMRT [10–13]. Among the five studies comparing the survival rates of X-ray IMRT and particle therapy [16, 17, 24, 26, 27], there is only one report of a multicenter randomized phase II trial [16], and in that trial, the primary endpoints were PFS and total toxicity burden (TTB: TTB was defined as adverse events that taken into account the type and severity of events). However, in the interim analysis of the trial, the TTB in the IMRT group was higher than that in the PBT group; therefore, the study was discontinued because the TTB exceeded the set threshold [16]. Furthermore, even though 72 patients were enrolled in IMRT and 73 in the PBT groups, there was no significant difference in the OS and PFS values among the 61 patients in the IMRT and 47 patients in the PBT groups included in their final analysis. Notably, three patients in their IMRT group died prematurely due to cardiopulmonary toxicity after chemoradiation, but none died in their PBT group [16]. In all these aforementioned five studies, the PBT group outperformed the IMRT group in terms of OS, even though one study [24] showed a statistically significant difference. Similar results were also obtained in their PFS tests.

The 3-year OS rate was calculated by extracting the data from our particle therapy cohort registry at the same stage as the clinical stage included in the three papers [3, 16, 17] that reported the OS and were evaluated by the *Z*-test (Table 3). The obtained results were then divided into good, poor and no differences, and no definite tendency was observed. Differences in treatment effects observed in this analysis may be due to differences in background distributions between studies, and we cannot make strong statements about differences in treatment effects. In fact, the distribution of clinical stages, which is assumed to affect prognosis, was different. Similarly, two studies [16, 17] reporting PFS in a likewise manner were compared with our studied particle therapy cohort (Table 3). Regarding the PFS, the results of the particle beam therapy registry were favorable in all cases; however, when compared with the report by Lin [16], we believed that a large number of stage I cases in the particle therapy registry affected the results. Furthermore, despite the fact that our results were significantly better than those of Fang [17], we believe that it is inappropriate to judge the superiority or inferiority of treatment methods by comparing them with reports from Europe and USA, which mainly target adenocarcinomas. Therefore, even though this study did not clarify the superiority of particle therapy over X-ray radiotherapy in terms of survival, we would like to continue this study through a reanalysis scheduled for 2023.

In patients with esophageal cancer, cardiopulmonary toxicity is one of the biggest problems post-radiotherapy [23]. Among the comparative studies of X-ray radiotherapy and particle therapy, three studies that reported adverse events by grade in IMRT, which had the best dose distribution, were reports from overseas. Therefore, we considered that because surgery was added in many cases in which a complete response was not obtained in the initial response, the frequency of adverse events after X-ray radiotherapy and particle therapy was higher than that reported in Japan [16, 24, 25]. However, the number of adverse events in the particle therapy group was low in both cases (Lin *et al*. [16]: 23 vs 13%, *P* = 0.19, Xi *et al*. [24]: 7.1 vs 3.0%, *P* = 0.17, Wang *et al*. [25]: 21 vs 13%, *P* = 0.053). In addition, Wang *et al*. described that the frequency of adverse events for PBT was significantly lower than that for X-ray radiotherapy in patients with cardiac dysfunction (32 vs 14%, *P* = 0.018) [25].

Cardiopulmonary adverse events have been suggested to be significantly reduced in patients with cervical esophageal cancer. From our results, we found that there were three cases (1.7%) and one case (0.6%) of cardiac and pulmonary adverse events of Grade 3 or higher. Our particle therapy cohort included 22 cases (12.6%) of cervical esophageal cancer cases, and when cervical esophageal cases were excluded, there were two cases (1.3%) and one case (0.7%) of cardiac and pulmonary adverse events of Grade 3 or higher. Comparing these with the results of the Japan Clinical Oncology Group (JCOG) study and Japanese Radiation Oncology Study Group (JROSG), regarding stage I esophageal cancer, and the frequency of Grade 3 or higher adverse events (heart: 1.4%, lung: 2.8%) in JCOG9708 [7], in which X-ray radiotherapy was performed only locally, omitting irradiation to the lymph node region, and the frequency of adverse events in JCOG0508 [8] (heart: 2.1%, lung: 1.0%), which administered a low dose of 41.4 Gy in most cases, the frequency of our particle therapy registry study was almost the same in the heart and slightly lower in the lung. However, in stages II and III advanced cancer, the frequency of occurrence in the particle beam registry was clearly lower than that in the JCOG and JROSG study [3–6], and the incidence of cardiopulmonary diseases increases with age. Therefore, because 44.1% of esophageal cancer patients are 70 years and above in Japan [2], it seems reasonable to reduce cardiopulmonary adverse events by using particle therapy.

This study has a limitation. We compared the survival rate of X-ray radiotherapy and that of particle therapy; therefore, we considered that the results might reflect the differences in background factors (number of cases for each clinical stage, histopathological type, etc.) rather than the superiority of the treatment methods.

In conclusion, particle therapy for esophageal cancer may reduce cardiopulmonary toxicity compared with X-ray radiotherapy. In our future study, we will reexamine survival in a reanalysis scheduled for 2023.

## DATA AVAILABILITY

The datasets analyzed during the current study are available from the corresponding author on reasonable request.

## PRESENTATION AT A CONFERENCE

This study was presented at JASTRO 2022. Clinical trial registration number: 28-7.
